# Retraction Note: Deep learning-accelerated image reconstruction in back pain-MRI imaging: reduction of acquisition time and improvement of image quality

**DOI:** 10.1007/s11547-026-02254-5

**Published:** 2026-06-26

**Authors:** Arne Estler, Till‑Karsten Hauser, Merle Brunnée, Leonie Zerweck, Vivien Richter, Jessica Knoppik, Anja Örgel, Eva Bürkle, Sasan Darius Adib, Holger Hengel, Konstantin Nikolaou, Ulrike Ernemann, Georg Gohla

**Affiliations:** 1 Diagnostic and Interventional Neuroradiology, Department of Radiology, University Hospital Tuebingen, Hoppe‑Seyler‑Str. 3, 72076 Tübingen, Baden‑Württemberg Germany; 2 Department of Neuroradiology, Neurological University Clinic, Heidelberg University Hospital, Im Neuenheimer Feld 400, 69120 Heidelberg, Germany; 3 Department of Neurosurgery, University of Tübingen, 72076 Tübingen, Germany; 4 Department of Neurology and Hertie‑Institute for Clinical Brain Research, University of Tübingen, 72076 Tübingen, Germany; 5 Department of Diagnostic and Interventional Radiology, University of Tuebingen, Hoppe‑Seyler‑Str. 3, 72076 Tuebingen, Germany

**Retraction Note to: La radiologia medica (2024) 129:478–487** **https://doi.org/10.1007/s11547-024-01787-x**

The authors hereby retract this article due to the inability to reproduce the reported findings and the unavailability of the original dataset.

The data initially used for the analysis cannot be accessed as a result of an unforeseeable backup failure. Subsequent efforts to verify and replicate the results identified only four patients meeting the stated inclusion criteria with a complete DRB protocol available in the PACS system. This number is substantially lower than that reported in the original manuscript, rendering the conclusions unreliable.

The authors extend their sincere apologies to the journal, its readership, and the scientific community for any inconvenience or misunderstanding caused by this publication.

The authors agree with this retraction.

